# Supplementary material for: Fecal Microbiota Transplantation from Mice Receiving Magnetic Mitohormesis Treatment Reverses High-Fat Diet-Induced Metabolic and Osteogenic Dysfunction
Source: Int J Mol Sci. 2025 Jun 6;26(12):5450. doi: 10.3390/ijms26125450 (PMC12193155; doi:10.3390/ijms26125450)
Supplement: Supplementary file 1 [file ijms-26-05450-s001.zip › ijms-3653820-supplementary.pdf]

Fecal Microbiota Transplantation from Mice Receiving Mag-netic Mitohormesis Treatment Reverses High-Fat Diet-Induced Metabolic and Osteogenic Dysfunction

Jun Kit Craig Wong [1,2,3, #], Bharati Kadamb Patel [1,#], Yee Kit Tai* [1,2,3,4,#], Tuan Zea Tan [5], Wei Wei Thwe Khine [6], Way Cherng Chen [7], Marek Kukumberg [8,9], Jianhong Ching [10,11], Lye Siang Lee [10], Kee Voon Chua [10], Tsze Yin Tan [10], Kwan Yu Wu [1,2,3,], Xizhe Bai [1,2,3], Jan Nikolas Iversen [1,2,3,] Kristy Purnamawati [1], Rufaihah Abdul Jalil [1,8,9,12], Alan Prem Kumar [4,13], Yuan Kun Lee [6], Shabbir M Moochhala [1,13], Alfredo Franco-Obregón* [1,2,3,4,8,14,15]

*These authors contributed equally to this work.

[#] Corresponding authors

Correspondence: alextai@nus.edu.sg (Y.K.T.); afo@nus.edu.sg (A.F.O.)

Supplementary Information

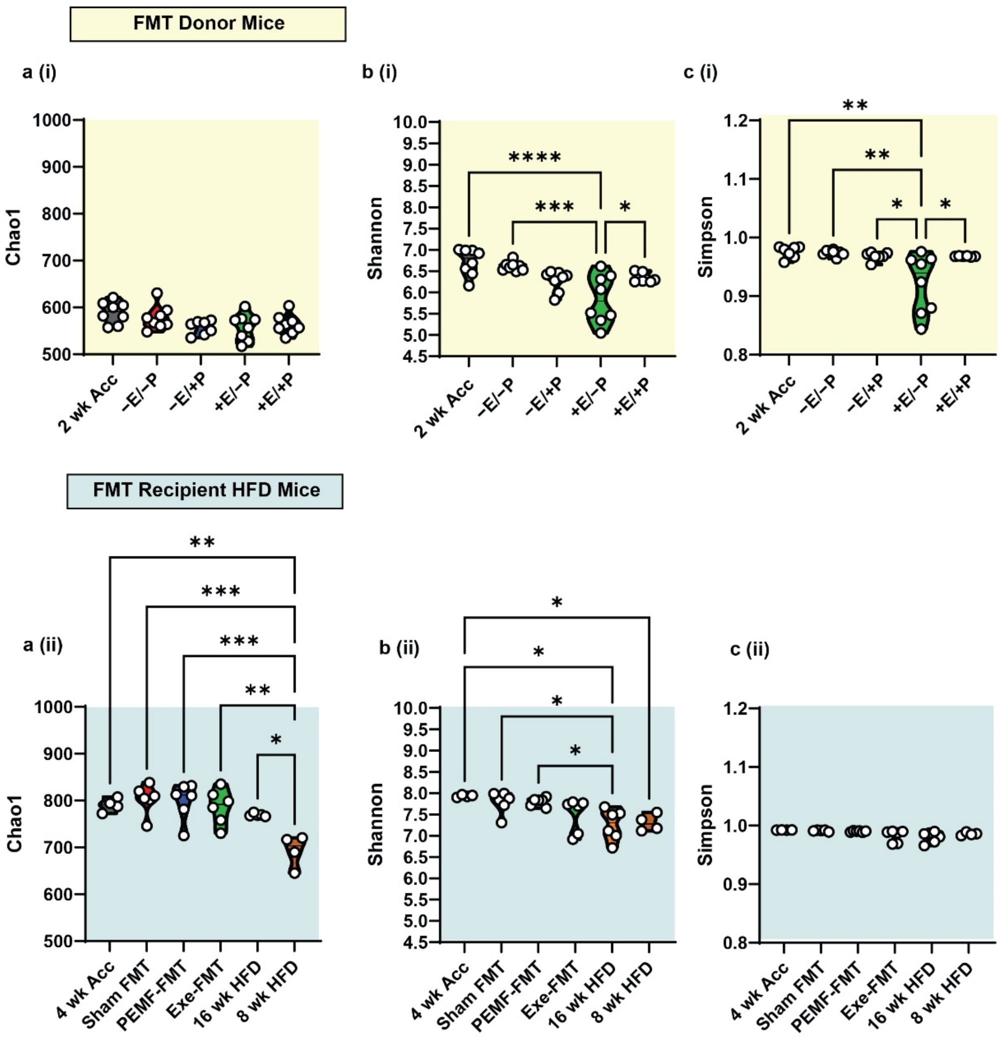

Figure S1. **Gut microbiota alpha diversity analysis in donor and recipient mice.** Microbial diversity was assessed using (**a**) Chao1 (richness), (**b**) Shannon (diversity), and (**c**) Simpson (dominance) indices, with (i) FMT-donor and (ii) FMT-recipient mice. Violin plots display individual mice (dots) of n=4-8 mice per group, with statistical comparisons by one-way ANOVA and Sidak's post hoc test (*$p<0.05$, **$p<0.01$). (E=exercise; P=PEMF).

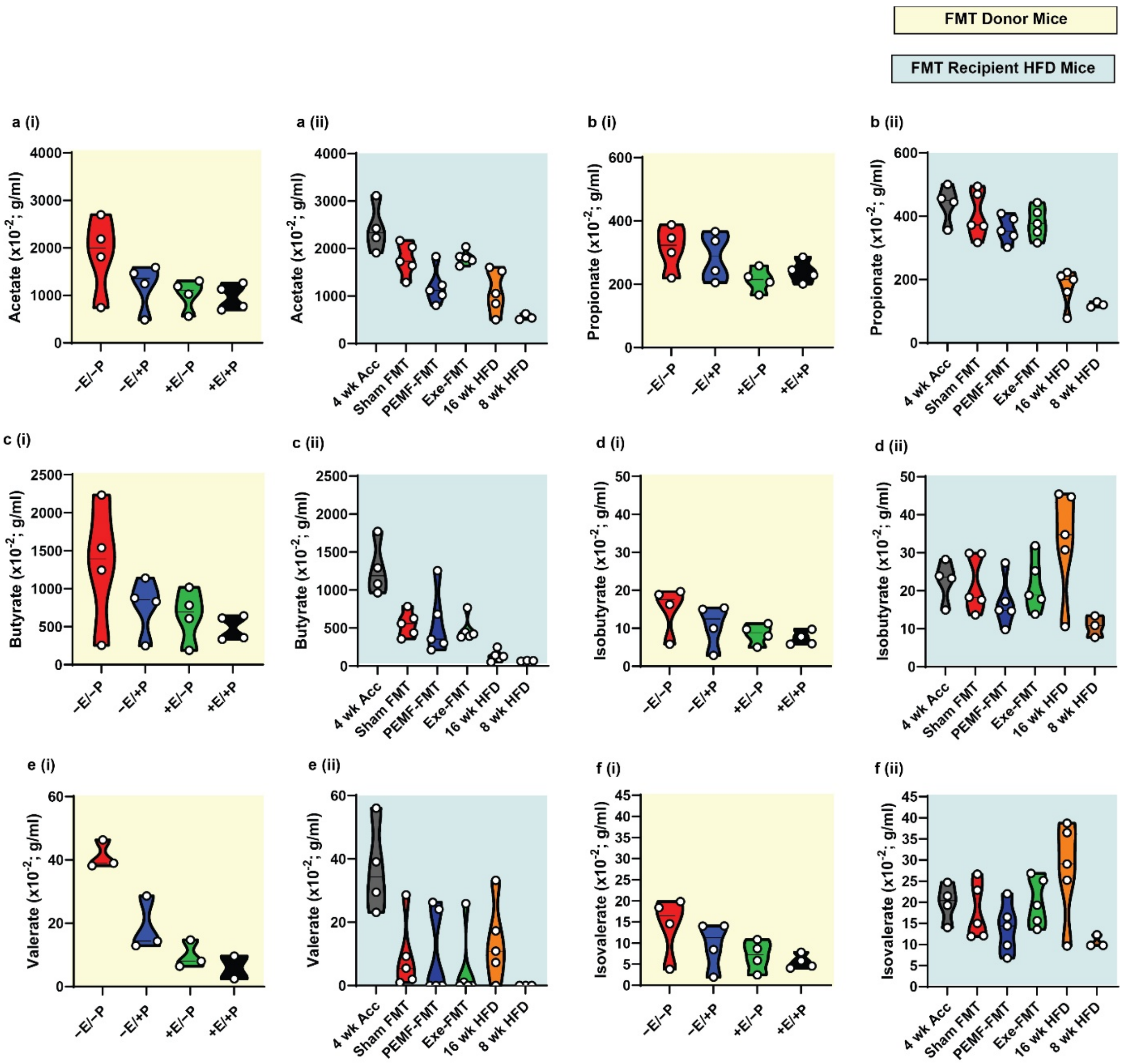

Figure S2. Short-chain fatty acid (SCFA) profiles in fecal samples. Concentrations (μg/mL per mg fecal matter) of (**a**) acetate, (**b**) propionate, (**c**) butyrate, (**d**) isobutyrate, (**e**) valerate, and (**f**) isovalerate were quantified in (i) FMT-donor and (ii) FMT-recipient mice. Violin plots display the average absolute concentration of individual SCFA. Each dot represents individual mice. Statistical significance was determined by one-way ANOVA and Sidak's post hoc test (*$p<0.05$, **$p<0.01$). (E=exercise; P=PEMF).

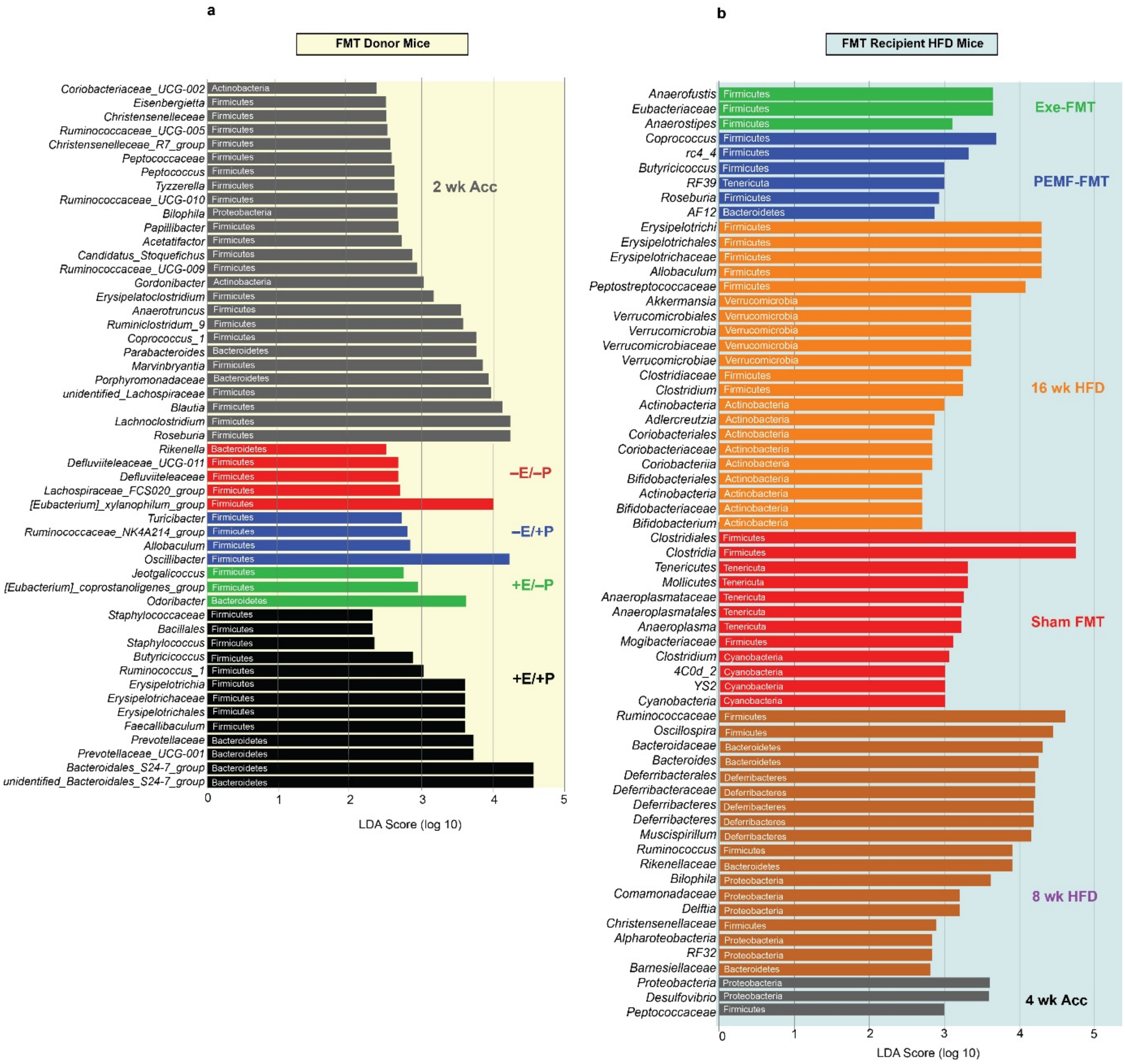

Figure S3. Fecal microbiota taxonomic profiling by LEfSe analysis. Linear discriminant analysis effect size (LEfSe) revealed significant taxonomic differences in gut microbiota between the different groups of (**a**) FMT-donor mice and (**b**) FMT-recipient HFD mice, with n=4-8 individual mice per group. LDA scores (log10) > 2 and $p < 0.05$ are shown. Cladograms illustrate the significantly differentially abundant operational taxonomic units (OTUs) between paired treatment groups. (E = exercise; P = PEMF).

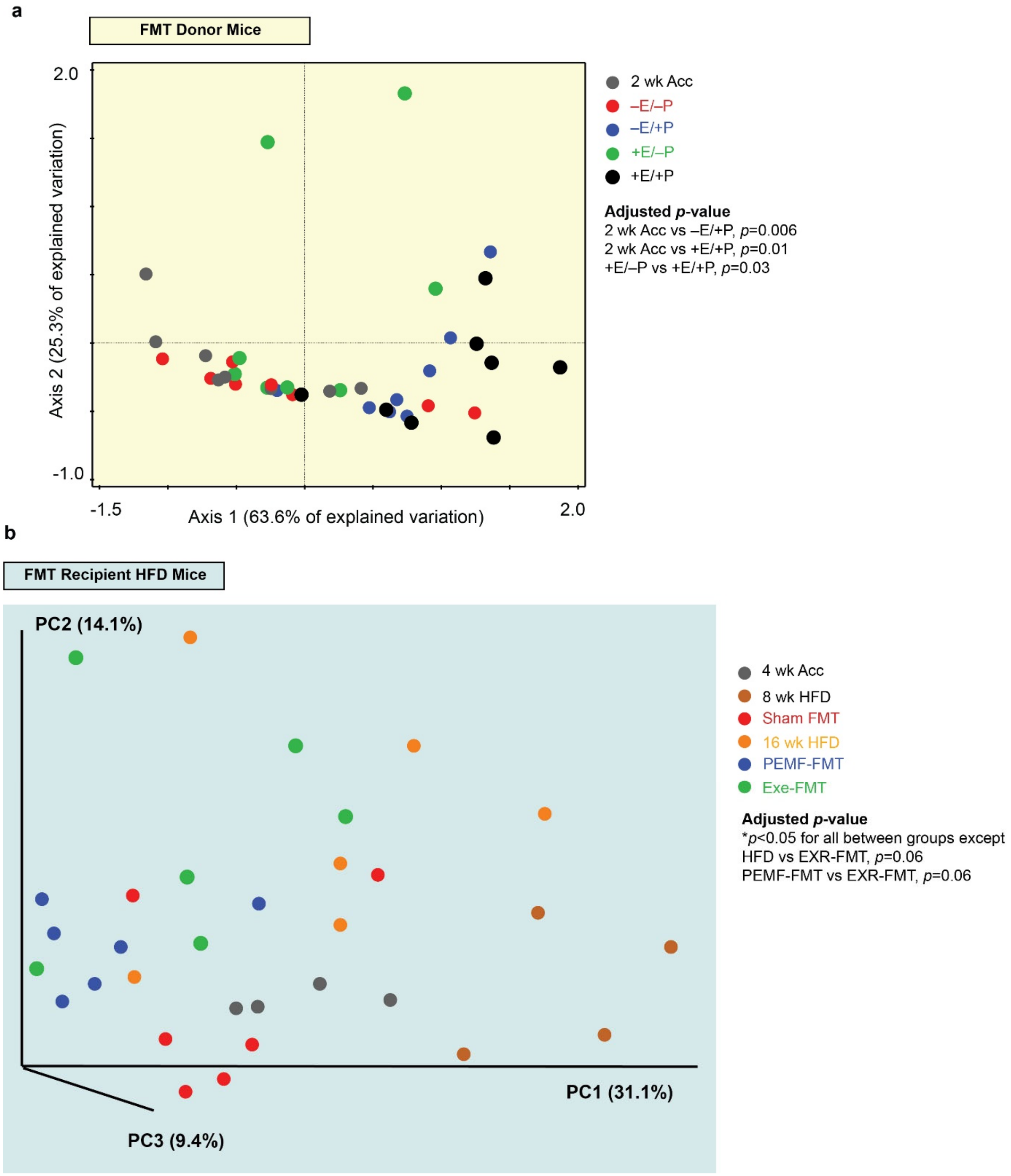

Figure S4. Principal Coordinate Analysis (PCO) of the microbial communities observed in fecal and cecal samples. Significant differences in OTU composition between treatments were found at adjusted *p*-values (≤0.05) using the PERMANOVA (Permutational Multivariate Analysis of Variance) test in (**a**) FMT Donor mice and (**b**) FMT-recipient HFD mice, with n=4-8 individual mice per group. (E = exercise; P = PEMF).

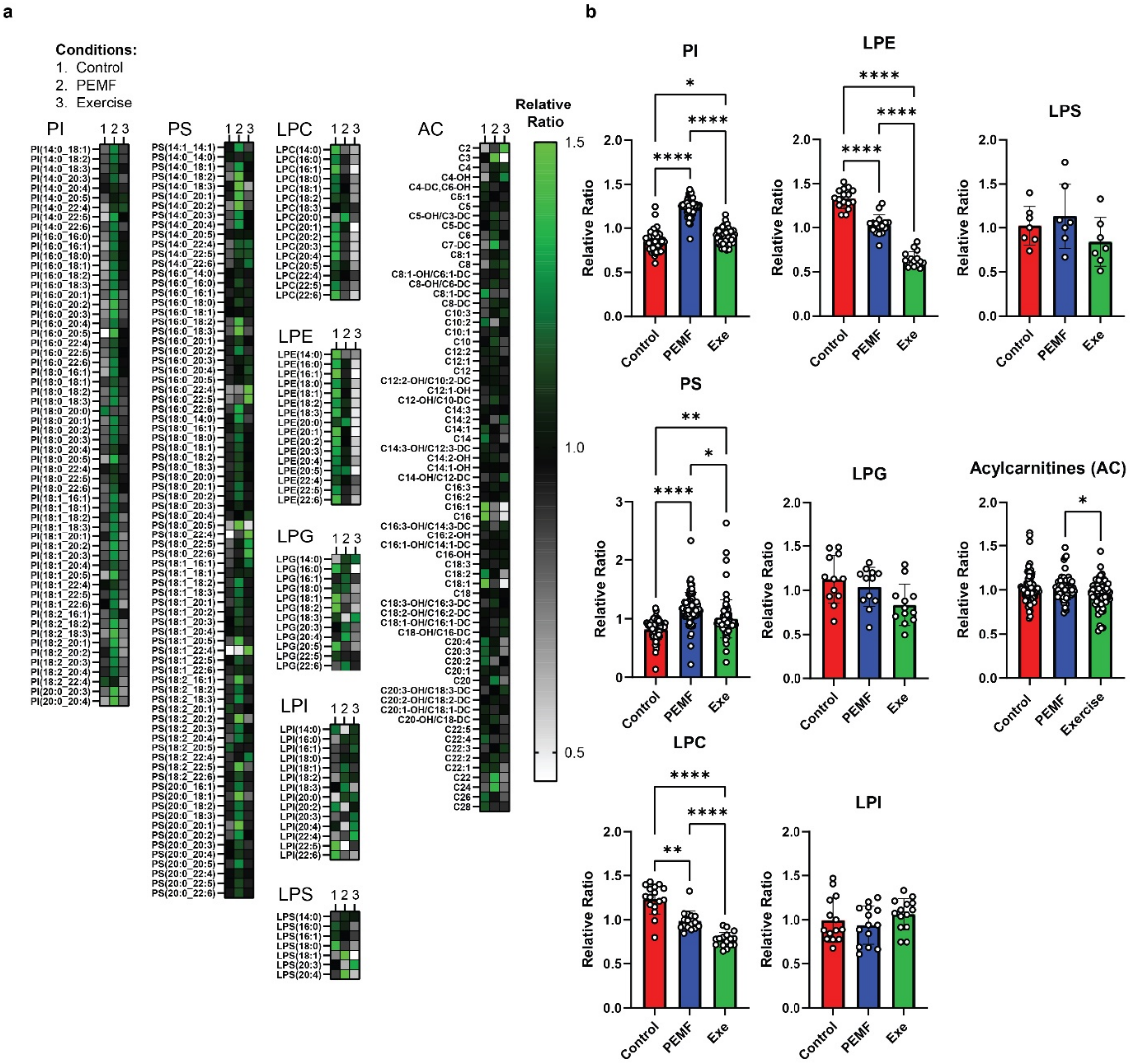

Figure S5. Hepatic lipid homeostasis modulation by PEMF exposure. LC-GC/MS analysis of liver lipids with each subspecies within a lipid class expressed as a relative ratio (n=5 mice per group). (**a**) Heatmap visualization of phospholipids (PI, PS), lysophospholipids (LPC, LPE, LPG, LPI, LPS) and acylcarnitine. (**b**) Bar chart summary of the average relative ratio of all lipid subspecies, with each point representing specific lipid molecules. Statistical analysis was determined using One-Way ANOVA with Tukey multiple comparisons test. Significant differences between treatment groups are denoted as *p<0.05, **p<0.01, ***p<0.001, and ****p<0.0001.

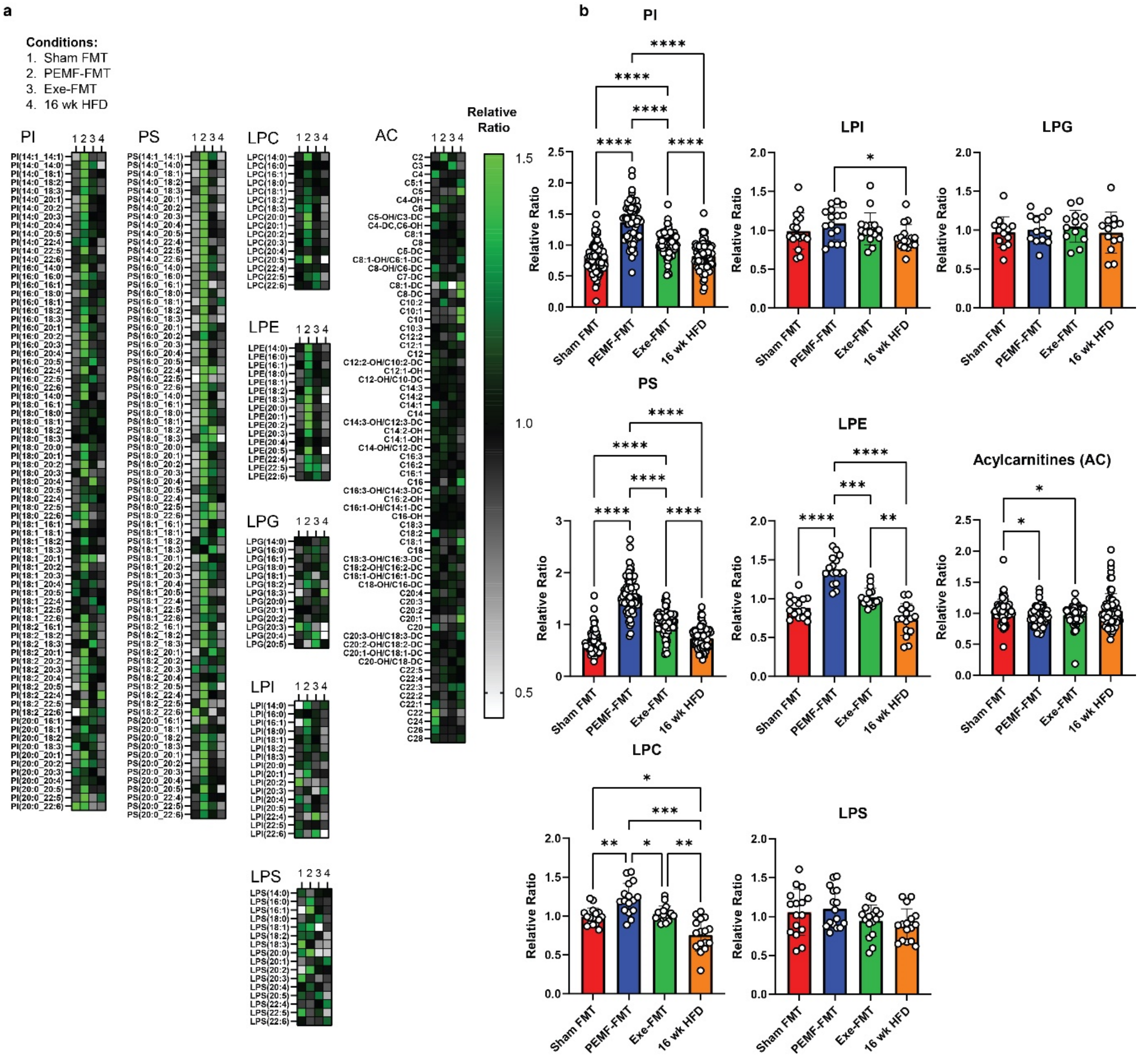

Figure S6. Modulation of hepatic lipid homeostasis in HFD mice in response to PEMF-FMT. LC-GC/MS analysis of liver lipids with each subspecies within a lipid class expressed as a relative ratio (n=4-6 mice per group). (**a**) Heatmap visualization of phospholipids (PI, PS), lysophospholipids (LPC, LPE, LPG, LPI, LPS) and acylcarnitine. (**b**) Bar chart summary of the average relative ratio of all lipid subspecies, with each point representing specific lipid molecules. Statistical analysis was determined using One-Way ANOVA with Tukey multiple comparisons test. Significant differences between treatment groups are denoted as *p<0.05, **p<0.01, ***p<0.001, and ****p<0.0001.

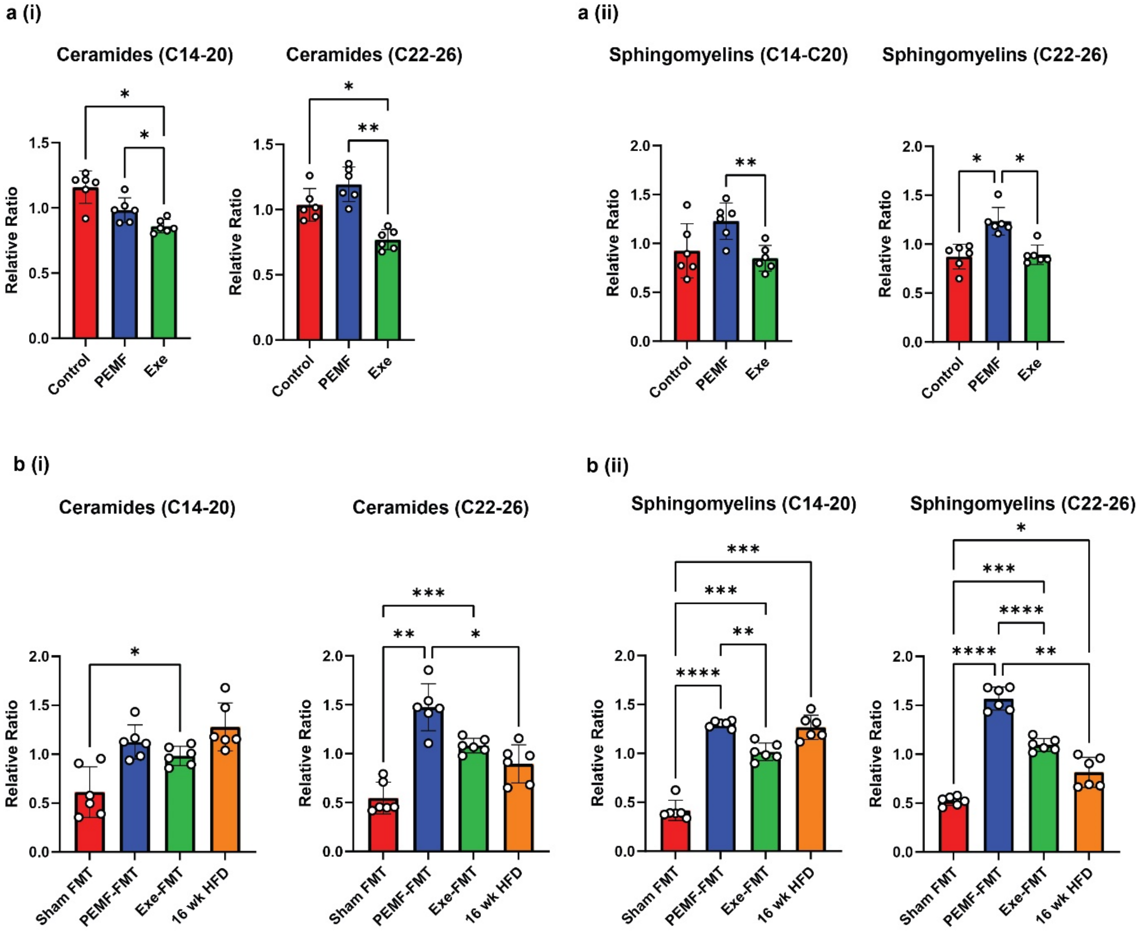

Figure S7. Sphingolipid remodeling in donor and recipient mice. Bar chart summary of the mean relative ratio of long-chain (C14-C20) and very long-chain (C22-C26) of (i) ceramides and (ii) sphingomyelins in (**a**) FMT-donor (n=5 mice per group) and (**b**) FMT-recipient mice (n=4-6 mice per group). Statistical analysis was performed by comparing the mean of relative ratios using One-Way ANOVA with Tukey multiple comparisons test. Significant differences between treatment groups are denoted as *p<0.05, **p<0.01, ***p<0.001, and ****p<0.0001.
